# Supplementary material for: Single-cell RNA sequencing reveals time- and sex-specific responses of mouse spinal cord microglia to peripheral nerve injury and links ApoE to chronic pain
Source: Nat Commun. 2022 Feb 11;13:843. doi: 10.1038/s41467-022-28473-8 (PMC8837774; doi:10.1038/s41467-022-28473-8)
Supplement: Supplementary file 1 — Supplementary information [file 41467_2022_28473_MOESM1_ESM.pdf]

Single-cell RNA sequencing reveals time- and sex-specific responses of mouse spinal cord microglia to peripheral nerve injury and links ApoE to chronic pain

Shannon Tansley[1,2‡], Sonali Uttam[1‡], Alba Ureña Guzmán[1‡], Moein Yaqubi[3], Alain Pacis[4], Marc Parisien[1,5], Haley Deamond[6], Calvin Wong[1], Oded Rabau[7], Nicole Brown[1], Lisbet Haglund[8], Jean Ouellet[7], Carlo Santaguida[9], Alfredo Ribeiro-da-Silva[5,6], Soroush Tahmasebi[10], Masha Prager-Khoutorsky[11], Jiannis Ragoussis[12,13], Ji Zhang[5,14], Michael W Salter[15], Luda Diatchenko[1,5], Luke M. Healy[3,*], Jeffrey S. Mogil[1,2,5*], Arkady Khoutorsky[1,5,*]

SUPLEMENTARY MATERIAL

Supplementary Figures

Legends for Supplementary Data

Supplementary References

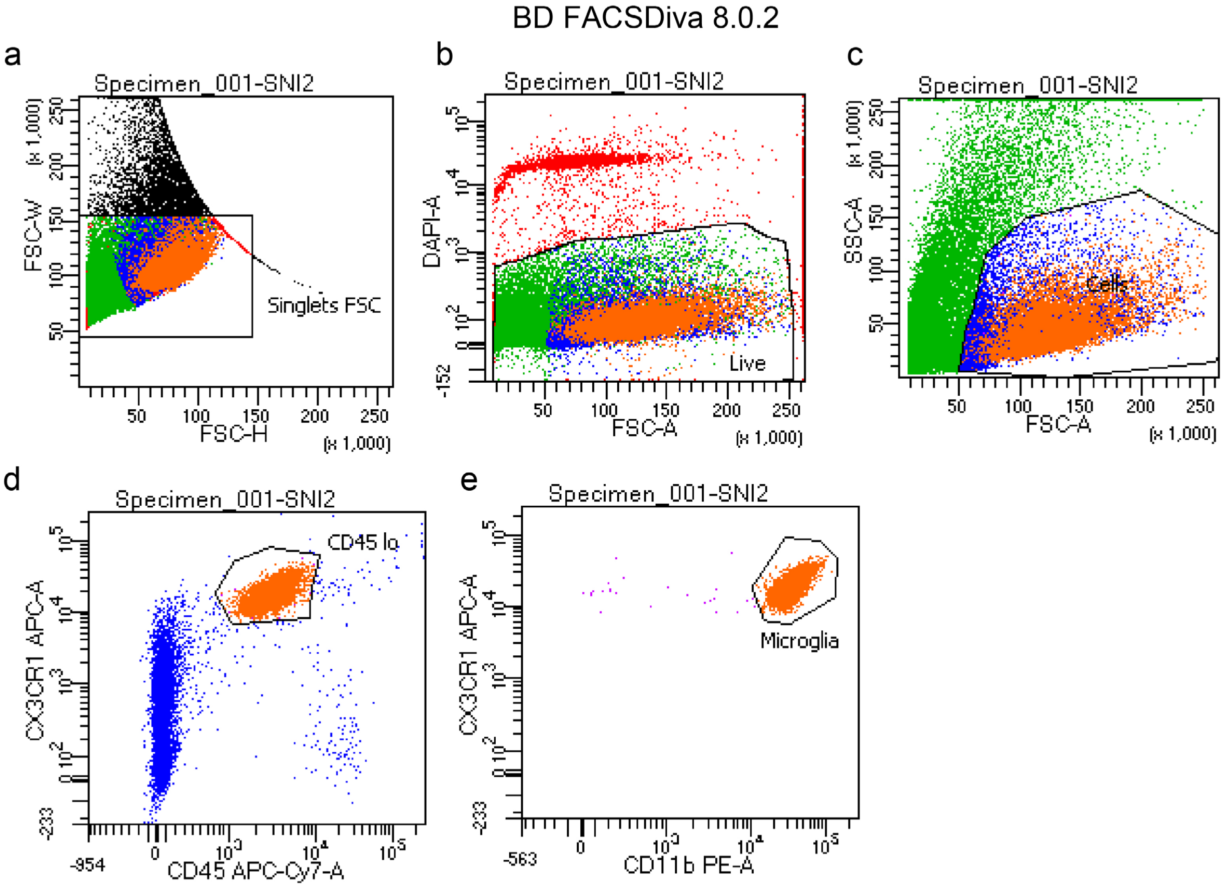

f

Tube: SNI2

Population	#Events	%Parent	%Total
All Events	51,779	####	100.0
Singlets FSC	45,787	88.4	88.4
Live	38,884	84.9	75.1
Cells	16,836	43.3	32.5
CD45 lo	6,913	41.1	13.4
Microglia	6,888	99.6	13.3

Supplementary Fig. 1. Fluorescence-activated cell sorting of mouse microglia.

a, Forward scatter of cells (y axis is height and x axis is width) to determine singlets. **b,** DAPI staining on y-axis, singlets on x-axis to determine live cells. Negative staining of DAPI are live cells. **c,** Side-scatter plotted against forward scatter to determine similarity of size and complexity of singlets. **d,** Singlets are carried forward with CX3CR1^{+} (high) staining and CD45 (low) staining. **e,** Singlets are carried forward with CX3CR1^{+} (high) staining and CD11b^{+} (high) staining. Microglial cells are subsequently sorted with these criteria. **f,** Microglia are 13.3% of all events.

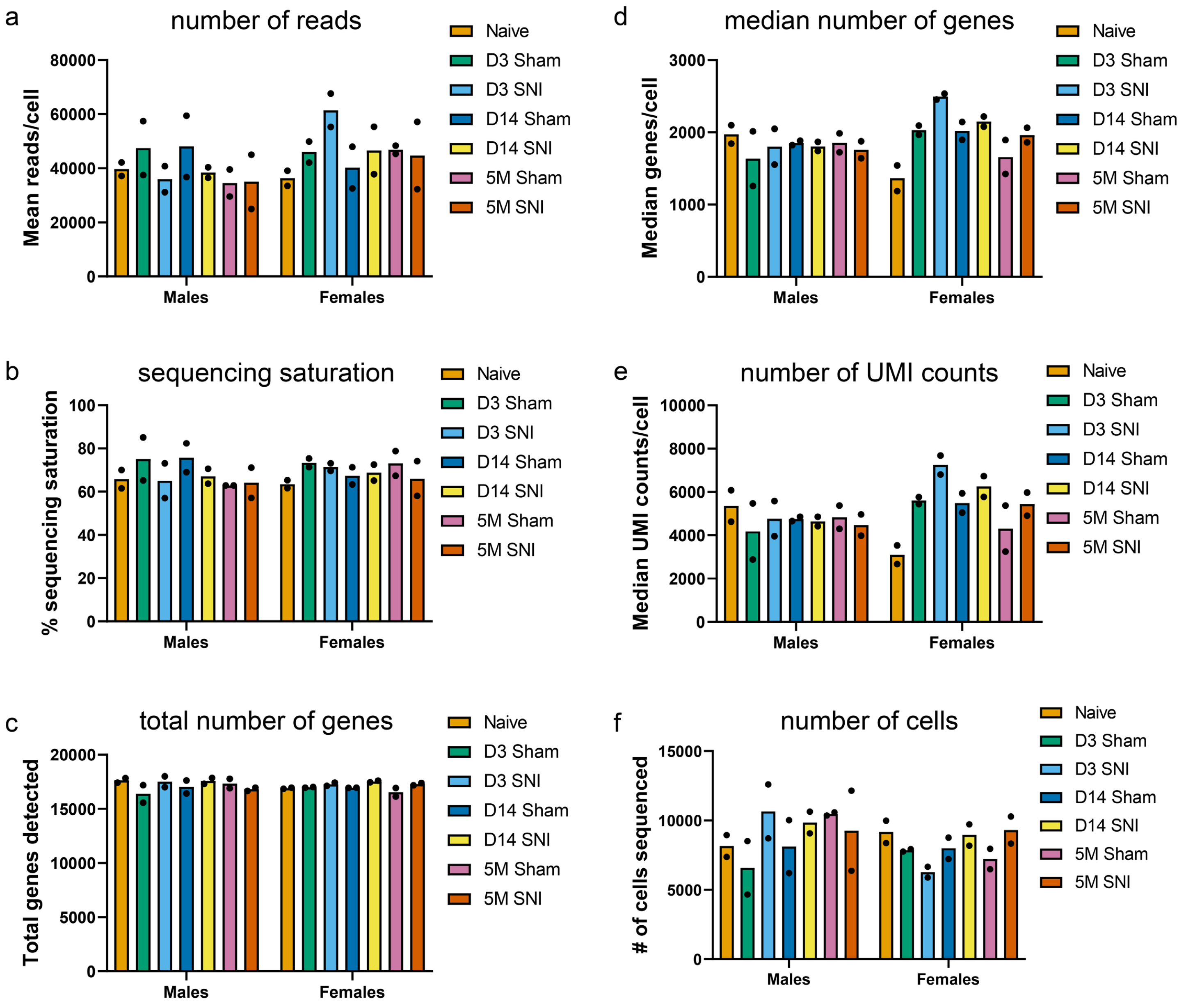

Supplementary Fig. 2. Sequencing metrics. Bar graphs showing metrics of all cells sequenced prior to filtering. **a,** Mean number of reads per cell by sample. **b,** Percentage of sequencing saturation by sample. **c,** Total number of genes detected per cell by sample. **d,** Median number of genes detected per cell by sample. **e,** Median number of UMI counts per cell by sample. **f,** Number of cells sequenced by sample estimated by Cell Ranger (10X Genomics).

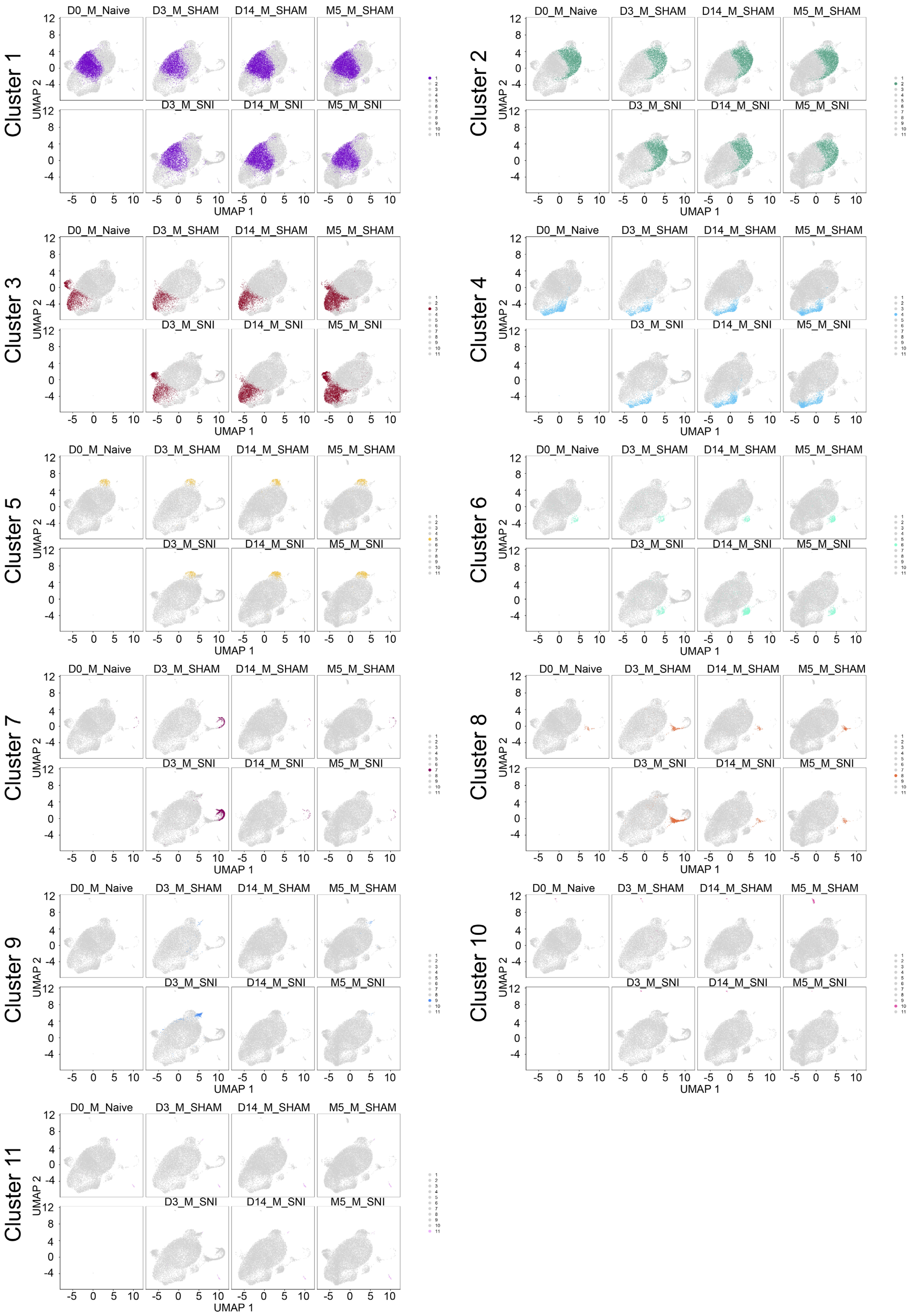

Cluster 1
Cluster 2
Cluster 3
Cluster 4
Cluster 5
Cluster 6
Cluster 7
Cluster 8
Cluster 9
Cluster 10
Cluster 11
D0_M_Naive
D3_M_SHAM
D14_M_SHAM
M5_M_SHAM
D3_M_SNI
D14_M_SNI
M5_M_SNI
UMAP 1
UMAP 2
12
8
4
0
-4
-5
0
5
10
1
2
3
4
5
6
7
8
9
10
11

Supplementary Fig. 3. UMAPs of 11 color-coded clusters in seven experimental conditions in males. D0_M_NAIVE - nave mice; D3_M_SHAM – Day 3 post-sham; D3_M_SNI – Day 3 post-SNI; D14_M_SHAM – Day 14 post-sham; D14_M_SNI – Day 14 post-SNI; M5_M_SHAM – 5 months post-sham; M5_M_SNI – 5 months post-SNI.

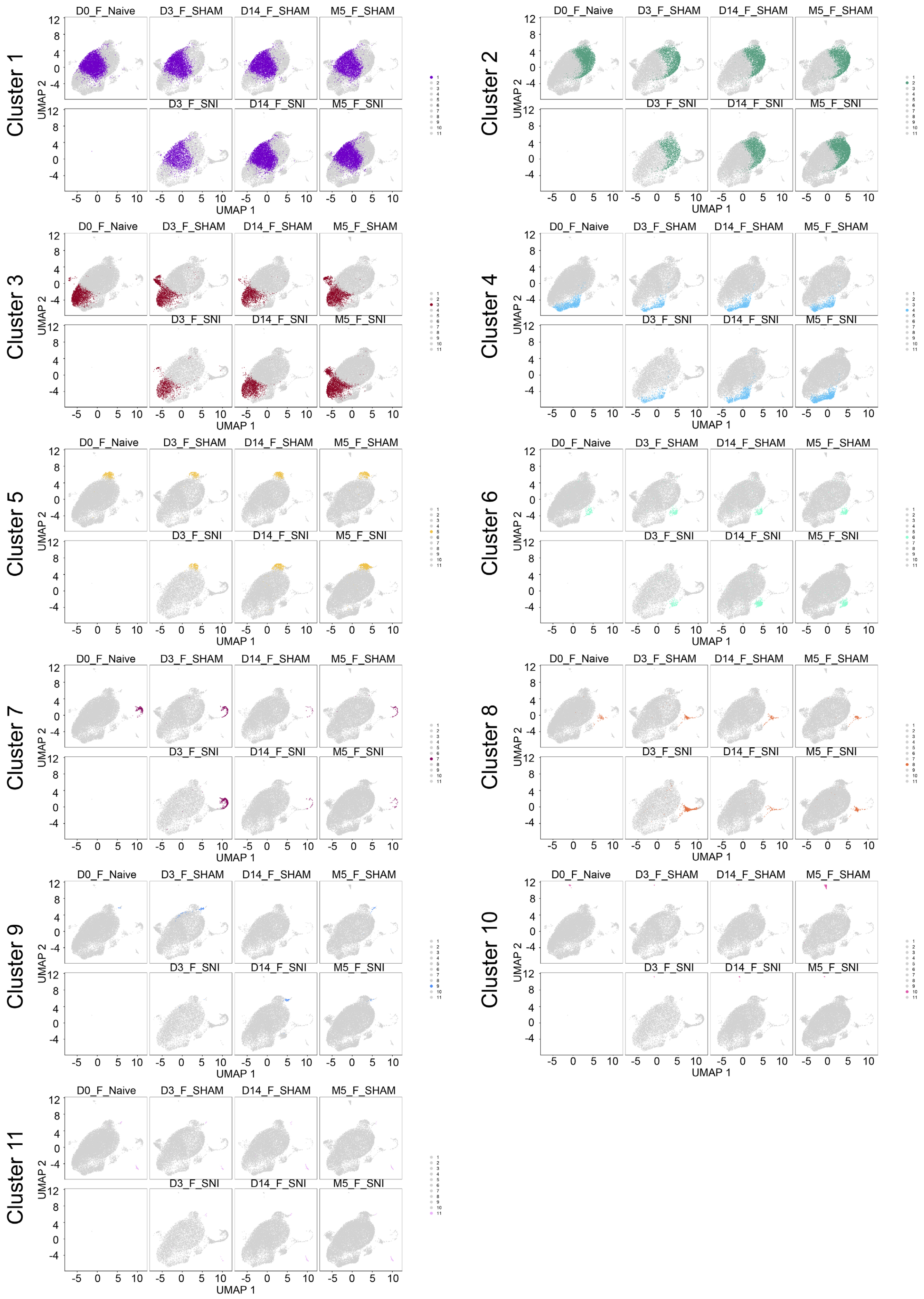
Cluster 1
Cluster 2
Cluster 3
Cluster 4
Cluster 5
Cluster 6
Cluster 7
Cluster 8
Cluster 9
Cluster 10
Cluster 11
D0_F_Naive
D3_F_SHAM
D14_F_SHAM
M5_F_SHAM
D3_F_SNI
D14_F_SNI
M5_F_SNI
UMAP 1
UMAP 2

Supplementary Fig. 4. UMAPs of 11 color-coded clusters in seven experimental conditions in females. D0_F_NAIVE - nave mice; D3_ F_SHAM – Day 3 post-sham; D3_F_SNI – Day 3 post-SNI; D14_F_SHAM – Day 14 post-sham; D14_F_SNI – Day 14 post-SNI; M5_F_SHAM – 5 months post-sham; M5_F_SNI – 5 months post-SNI.

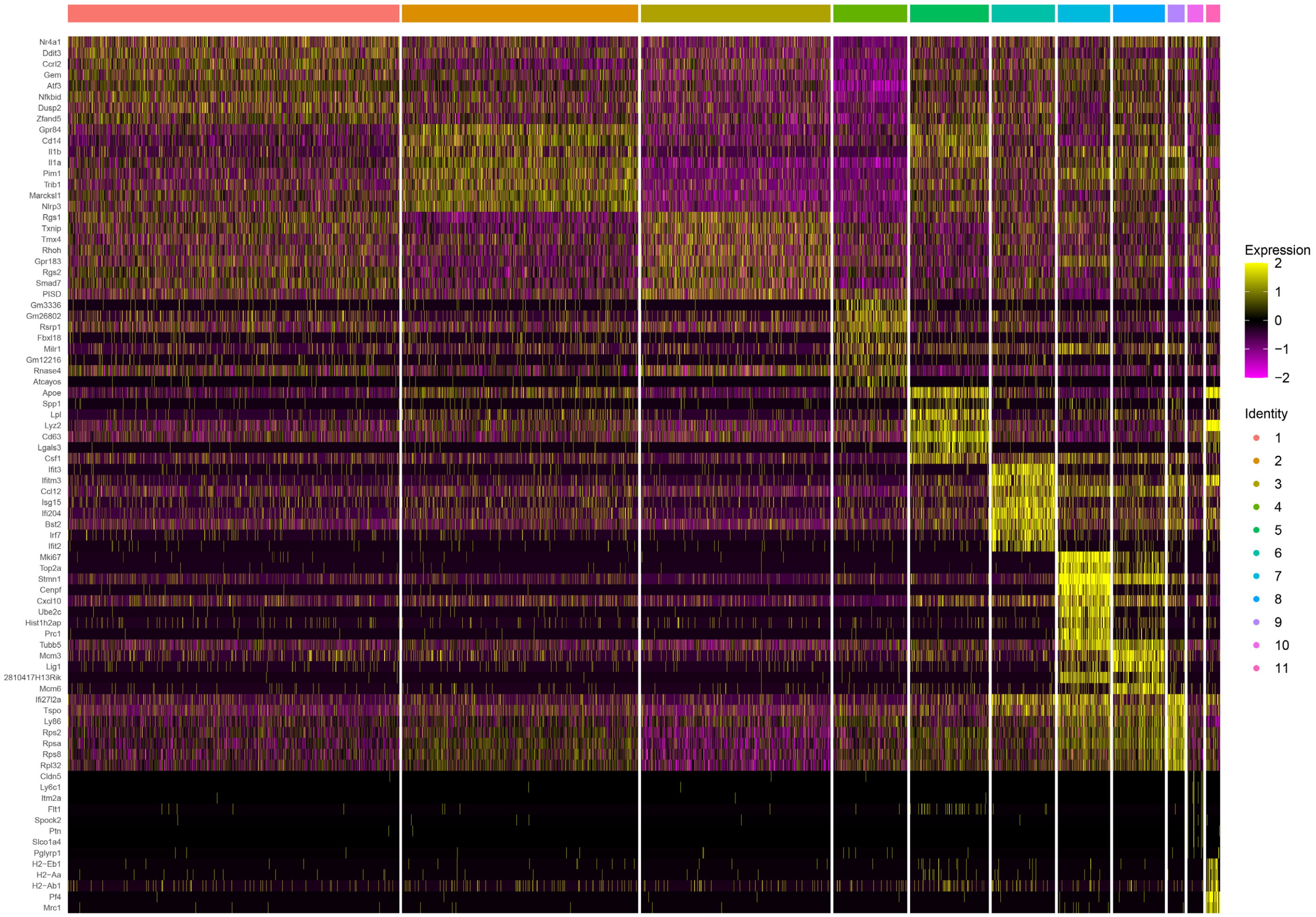

Supplementary Fig. 5. Heatmap showing expression of top 8 highly expressed genes in each cluster. Expression of genes is color-coded, where yellow indicates higher expression and purple indicates lower expression. See Supplementary Data 2 for a complete list of clusters markers.

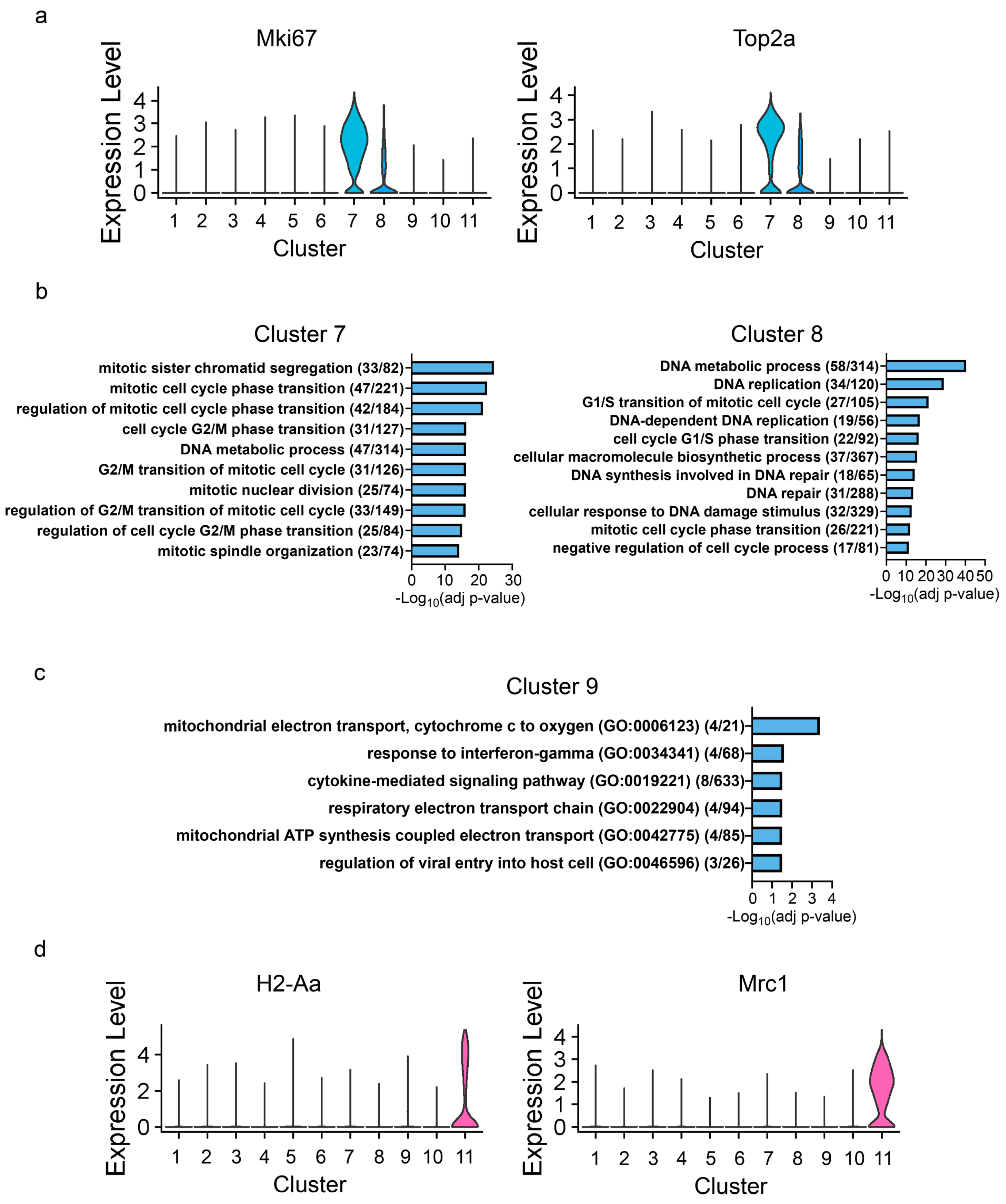
a
Mki67
Top2a
Expression Level
Cluster
b
Cluster 7
mitotic sister chromatid segregation (33/82)
mitotic cell cycle phase transition (47/221)
regulation of mitotic cell cycle phase transition (42/184)
cell cycle G2/M phase transition (31/127)
DNA metabolic process (47/314)
G2/M transition of mitotic cell cycle (31/126)
mitotic nuclear division (25/74)
regulation of G2/M transition of mitotic cell cycle (33/149)
regulation of cell cycle G2/M phase transition (25/84)
mitotic spindle organization (23/74)
-Log10(adj p-value)
Cluster 8
DNA metabolic process (58/314)
DNA replication (34/120)
G1/S transition of mitotic cell cycle (27/105)
DNA-dependent DNA replication (19/56)
cell cycle G1/S phase transition (22/92)
cellular macromolecule biosynthetic process (37/367)
DNA synthesis involved in DNA repair (18/65)
DNA repair (31/288)
cellular response to DNA damage stimulus (32/329)
mitotic cell cycle phase transition (26/221)
negative regulation of cell cycle process (17/81)
-Log10(adj p-value)
c
Cluster 9
mitochondrial electron transport, cytochrome c to oxygen (GO:0006123) (4/21)
response to interferon-gamma (GO:0034341) (4/68)
cytokine-mediated signaling pathway (GO:0019221) (8/633)
respiratory electron transport chain (GO:0022904) (4/94)
mitochondrial ATP synthesis coupled electron transport (GO:0042775) (4/85)
regulation of viral entry into host cell (GO:0046596) (3/26)
-Log10(adj p-value)
d
H2-Aa
Mrc1
Expression Level
Cluster

Supplementary Fig. 6. Gene expression and GO analysis show heterogeneous transcriptional states in spinal microglia. a, Violin plots showing the expression of *Mki67* and *Top2a* transcripts across clusters. GO terms (biological process) enriched in markers from clusters 7 and 8 **(b)**, and the top 40 markers from cluster 9 **(c).** Analyses in **b** and **c** were performed using Enrichr (employing Fisher's exact test), p-values were corrected for multiple testing using the Benjamini-Hochberg method. **d,** Violin plots showing the expression of *H2-Aa* and *Mrc1* transcripts across clusters.

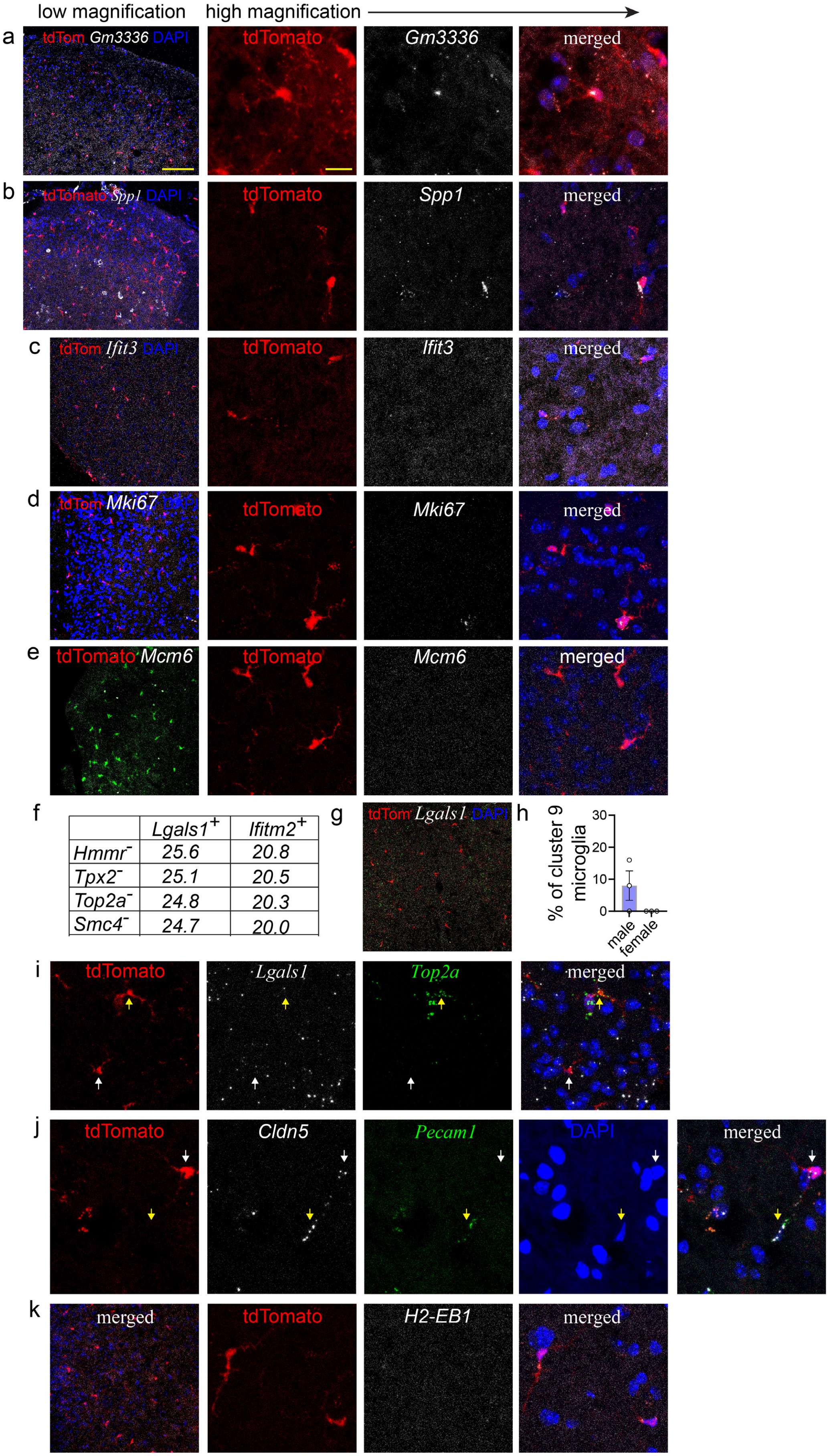

	$Lgals1^{+}$	$Ifitm2^{+}$
$Hmmr^{-}$	25.6	20.8
$Tpx2^{-}$	25.1	20.5
$Top2a^{-}$	24.8	20.3
$Smc4^{-}$	24.7	20.0

Supplementary Fig. 7. Identification of specific microglia clusters in the dorsal horn spinal cord using *in situ* hybridization. TdTomato;TMEM119^{CreERT2} mice were used to visualize microglia in the spinal cord dorsal horn. Spinal cord sections from mice at day 3 post-SNI were processed for *in situ* hybridization (using RNAscope) and the images were collected using confocal microscopy. Unique cluster markers were used to identify microglia belonging to cluster 4 (**a**, *Gm3336),* 5 (**b**, *Spp1*$^+$), 6 (**c**, *Ifit3*$^+$), 7 (**d**, *Mki67*$^+$), 8 (**e**, *Mcm6*$^+$), 10 (**j**, *Cldn5*$^+$), and 11 (**k**, *H2-EB1*$^+$). Eight best combinations of two markers to identify cluster 9 microglia are shown in **f**. The percent of cluster 9 microglia that can be identified by a combination of two markers is shown. *In situ* hybridization for *Lgals1* and *Top2a* reveals microglia belonging to cluster 9 (**g**, low magnification, **i**, high magnification, white arrow, *Lgals1*$^+$*Top2a*$^-$) and likely proliferating microglia (**i**, yellow arrow, *Lgals1*$^-$*Top2a*$^+$). **h**, The number of cluster 9 microglia at day 3 post-SNI in males and females was calculated by counting the proportion of *Lgals1*$^+$*Top2a*$^-$ microglia (tdTomato-positive) and correcting for the fraction of cluster 9 microglia that can be detected using *Lgals1*$^+$*Top2a*$^-$ markers as shown in **f.** N=3 mice/group. Data are presented as mean ± s.e.m. **j**, Vessel-associated microglia (white arrow) were identified by the presence of *Cldn5* and the absence of the endothelial marker *Pecam1*. Yellow arrow marks *Cldn5*$^+$*Pecam*$^+$ cell, likely representing endothelial cells (note the different shape of the nucleus in blue). Scale bar is 100 μm for low magnification and 10 μm for high magnification images.

a

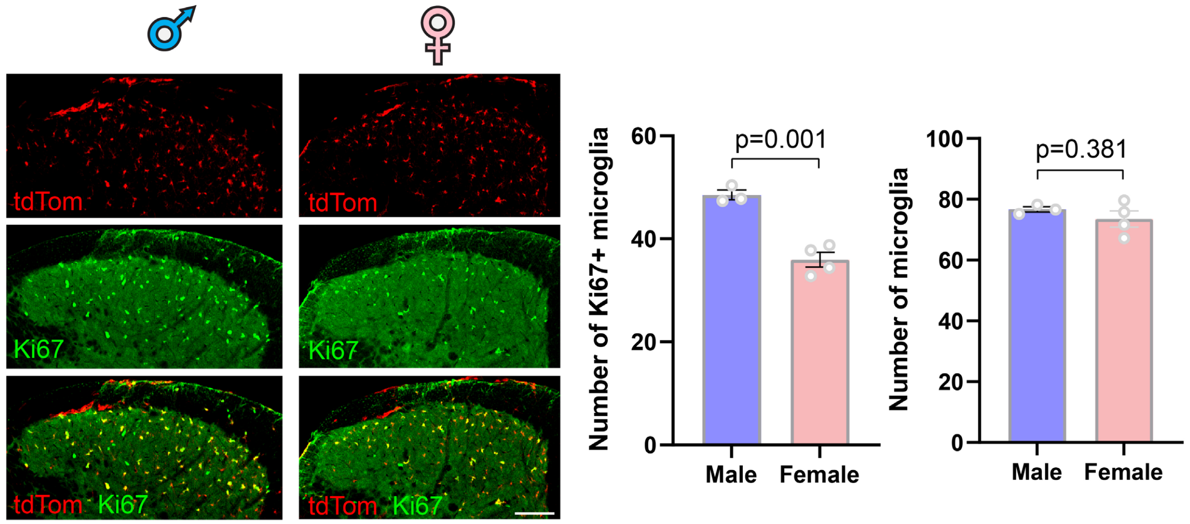

b

		Upregulated Rpl genes						Upregulated Rps genes						Downregulated Rpl genes						Downregulated Rps genes					
Sex	Cluster / Time point	1	2	3	4	5	6	1	2	3	4	5	6	1	2	3	4	5	6	1	2	3	4	5	6
Males	3 days post-SNI	7	0	15	10	3	14	5	2	17	5	3	16	0	1	0	0	0	0	1	2	0	0	2	0
	14 days post-SNI	0	0	0	0	2	6	0	0	0	0	2	2	0	0	0	0	0	0	0	0	0	0	0	1
	5 months post-SNI	0	0	0	0	2	0	0	0	0	0	4	0	15	16	12	16	9	11	8	9	5	12	5	4
Females	3 days post-SNI	0	0	0	0	0	0	0	0	0	0	0	1	0	0	0	0	0	0	0	0	0	0	0	0
	14 days post-SNI	0	0	0	0	0	0	1	0	1	0	0	1	0	0	0	0	0	0	0	0	0	0	0	0
	5 months post-SNI	0	0	0	0	1	1	0	0	0	0	0	0	0	0	0	0	0	0	0	0	1	0	0	0

Supplementary Fig. 8. Peripheral nerve injury induces changes in the expression of ribosomal proteins. a, Male and female mice expressing tdTomato under the microglia-specific promoter, TMEM119 (TdTomato;TMEM119^{CreERT2}), were subjected to the spared nerve injury (SNI) and the spinal cord tissue was collected at day 3 post-SNI (males n=3 mice/group, females n=4 mice/group). Scale bar is 100 μm. An unpaired two-tailed t-test was used to compare two groups. Data are plotted as mean ± s.e.m. **b**, Number of mRNAs encoding ribosomal proteins showing differential expression across conditions. Small ribosomal subunit (Rps), large ribosomal subunit (Rpl).

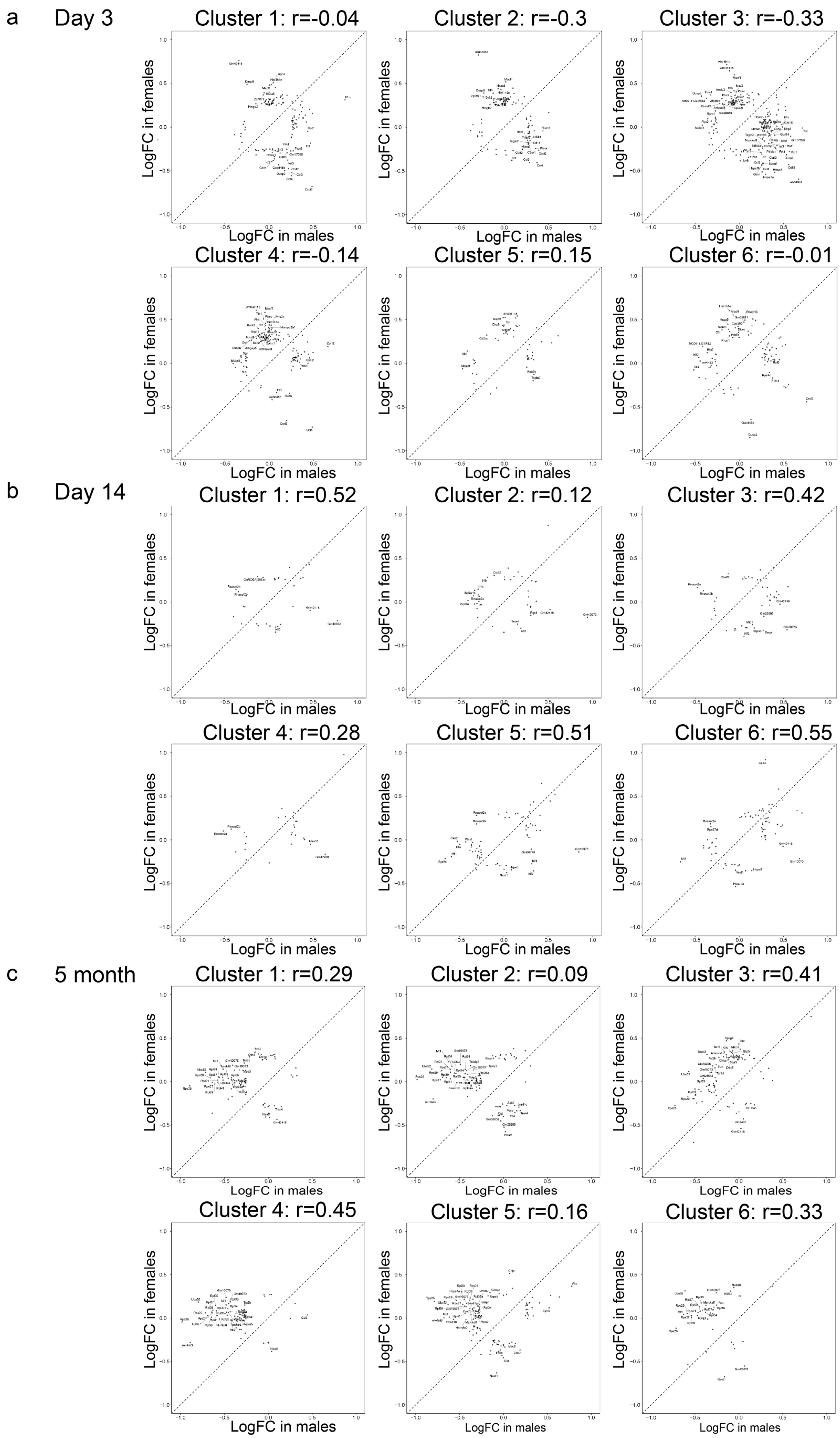
a
Day 3
Cluster 1: r=-0.04
Cluster 2: r=-0.3
Cluster 3: r=-0.33
Cluster 4: r=-0.14
Cluster 5: r=0.15
Cluster 6: r=-0.01
b
Day 14
Cluster 1: r=0.52
Cluster 2: r=0.12
Cluster 3: r=0.42
Cluster 4: r=0.28
Cluster 5: r=0.51
Cluster 6: r=0.55
c
5 month
Cluster 1: r=0.29
Cluster 2: r=0.09
Cluster 3: r=0.41
Cluster 4: r=0.45
Cluster 5: r=0.16
Cluster 6: r=0.33
LogFC in females
LogFC in males

Supplementary Fig. 9. Correlation between changes in gene expression in male and female microglia. Correlational analysis between differentially expressed genes (DEGs) in male and female microglia in each of the first six clusters on day 3 **(a),** day 14 **(b)** and 5 months **(c)** post-SNI. Pearson correlation coefficients (r) between males and females were calculated using the log fold change (logFC) values of genes that are differentially expressed in either sex.

Time point		Day 3		Day 14		Month 5	
Gene	Cluster	Males	Females	Males	Females	Males	Females
Dusp1	2			0.266 (3.16E-55)			
	3	-0.342 (1.069E-28)					
	5			0.368 (3.52E-10)			
	6			0.296 (0.007)			-0.269 (4.95E-03)
	9	-0.523 (0.015)					
Dusp6	1		0.490 (4.26E-93)				
	2		0.390 (2.04E-33)				
	3	-0.428 (1.07E-26)	0.354 (7.73E-21)				
	4		0.261 (5.16E-03)				
	7		0.341 (0.010)				
	8	-0.386 (1.141E-04)					
Il1b	1	0.861 (1.40E-59)	0.311 (5.46E-16)		0.280 (7.10E-14)		
	2	0.504 (1.57E-12)			0.282 (2.84E-03)		
	3	0.531 (3.74E-07)	0.449 (2.86E-04)				
	7	0.926 (1.61E-11)					
	8	0.847 (3.21E-06)					
	9	1.70 (3.78E-05)					
Tnf	1	0.471 (4.02E-77)					
	2	0.280 (3.87E-09)			0.292 (2.81E-20)		
	3	0.711 (6.77E-47)					
	6	0.552 (0.008)					
	7	0.537 (5.29E-15)					
	8	0.570 (1.63E-14)					
	9	0.879 (5.67E-08)					
Tspo	1	0.375 (1.29E-69)	0.295 (2.59E-47)				
	2		0.267 (1.02E-23)				
	3	0.419 (1.13E-30)	0.366 (4.63E-21)				
	4	0.376 (3.56E-09)	0.319 (5.16E-05)				
	5	0.366 (0.015)	0.405 (0.001)				
	6			0.486 (2.96E-06)			
	8	0.286 (1.07E-07)		0.698 (0.029)			
	9	0.639 (2.32E-06)					

Supplementary Fig. 10. Expression changes in MAPK and inflammation related genes. Table showing average log-transformed fold changes of MAPK-specific phosphatases *Dusp1* and *Dusp6*, pro-inflammatory genes *Il1b* and *Tnf*, and microglia activation marker *Tspo*. Upregulated genes are shown in red and downregulated genes in blue. Adjusted p-values are shown in brackets. Differentially expressed genes were calculated using the Wilcoxon rank-sum test (two-sided) with Bonferroni correction.

a

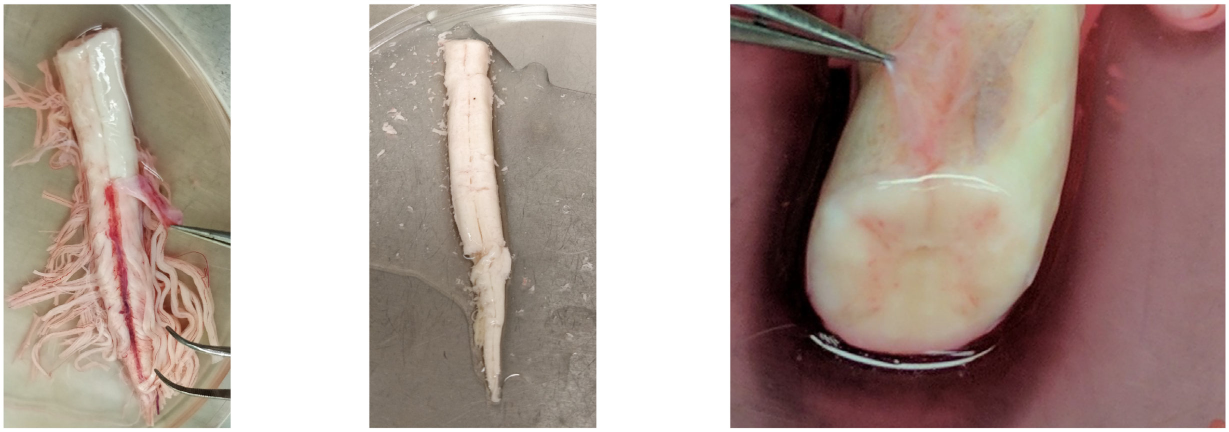

b

Sample	Sex	Age	Number of cells	Mean reads per cell	Mean genes per cell	UMI counts	Sequencing saturation
F1	Female	39-68	1394	232782	1570	15809	93.8
F2	Female	38-68	5510	61666	2453	19678	70.5
M1	Male	39-68	3211	107218	1538	18460	86.8

c

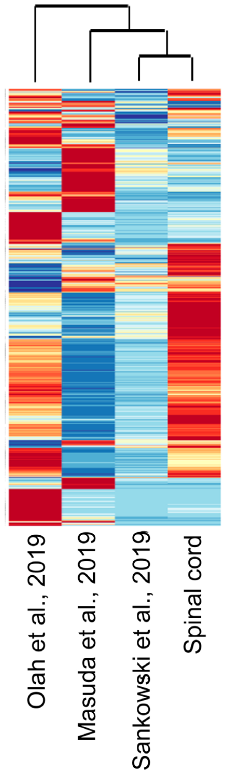

d

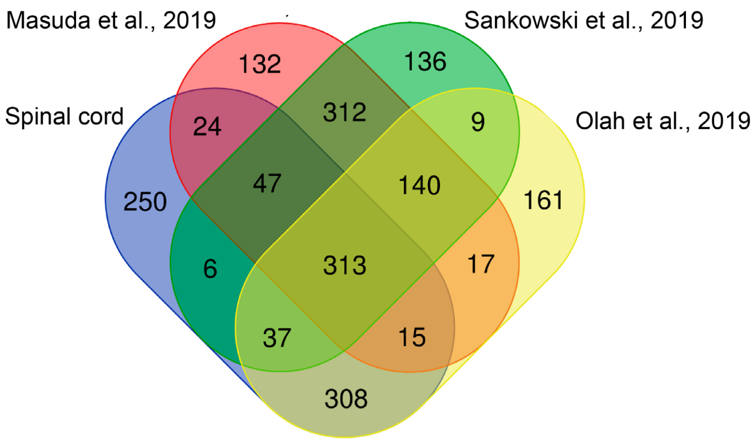

e

	Library Preparation	Sequencing Platform	Sequencing Depth	#Cells Sequenced	Tissue Source
Spinal Cord	10X Chromium	HiSeq 4000	60,000 - 230,000 reads per cell	5,456	Rapid Autopsy
Sankowski et al., 2019	mCEL-Seq2	HiSeq 3000	130,000 - 200,000 reads per cell	4,396	Surgical Resection
Masuda et al., 2019	Smart-Seq2	HiSeq 3000	~200,000 reads per cell	1,180	Surgical Resection
Olah et al., 2020	10X Chromium	HiSeq 4000	66,000 - 1,121,969 reads per cell	16,242	Mixture Autopsy/Surgical

f g h

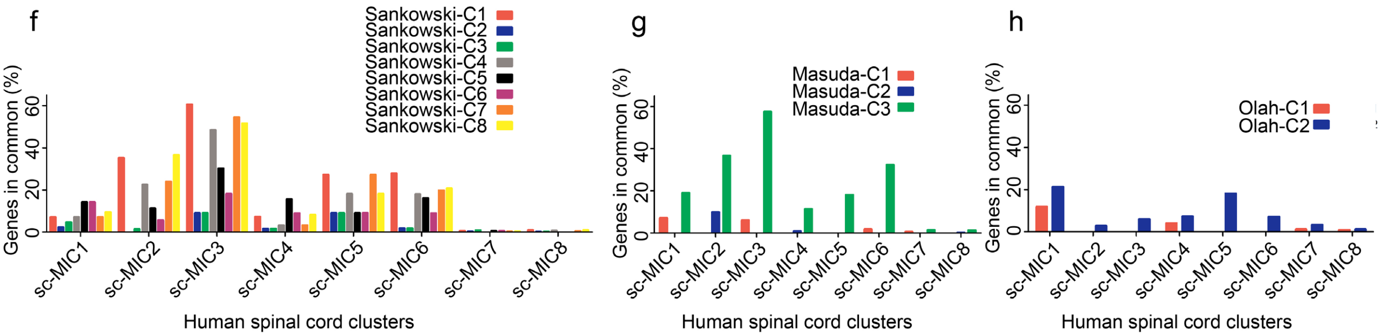

Supplementary Fig. 11. Information on rapid autopsy human spinal cord material. a, Rapid autopsy human spinal cord tissue is provided from the level of the first lumbar nerve (L1) caudally to the *conus terminalis*. Meningeal layer is removed in preparation of tissue for digestion. **b,** Donor demographics and sequencing parameters. **c**, A heatmap showing the expression of the top 1000 highly expressed genes in our human spinal cord microglia dataset and the expression of these genes in non-spinal cord (brain) microglia in 3 publicly available studies: (Masuda et al.,[1]), (Sankowski et al., [2]), and (Olah et al., [3]). The gene list with expression values of the analysed 1000 genes can be found in Supplementary Data 9. **d**. A Venn diagram using the top 1000 highly expressed genes in each dataset. **e**. A table showing basic metrics of the used datasets including library preparation technology, sequencing platform and depth, number of sequenced cells and the source of the tissue. **f-h**. The similarity of genes expressed in spinal cord (current study) and brain (Masuda et al.,[1], Sankowski et al., [2], and Olah et al., [3]) microglia was studied on a per-cluster basis. Bar plots depicting the percentage of common upregulated genes between spinal cord (sc-MIC) and brain microglia clusters from 3 publicly available studies: Sankowski et al., [2] (**f**), Masuda et al.,[1] (**g**) and Olah et al., [3] (**h**) respectively.

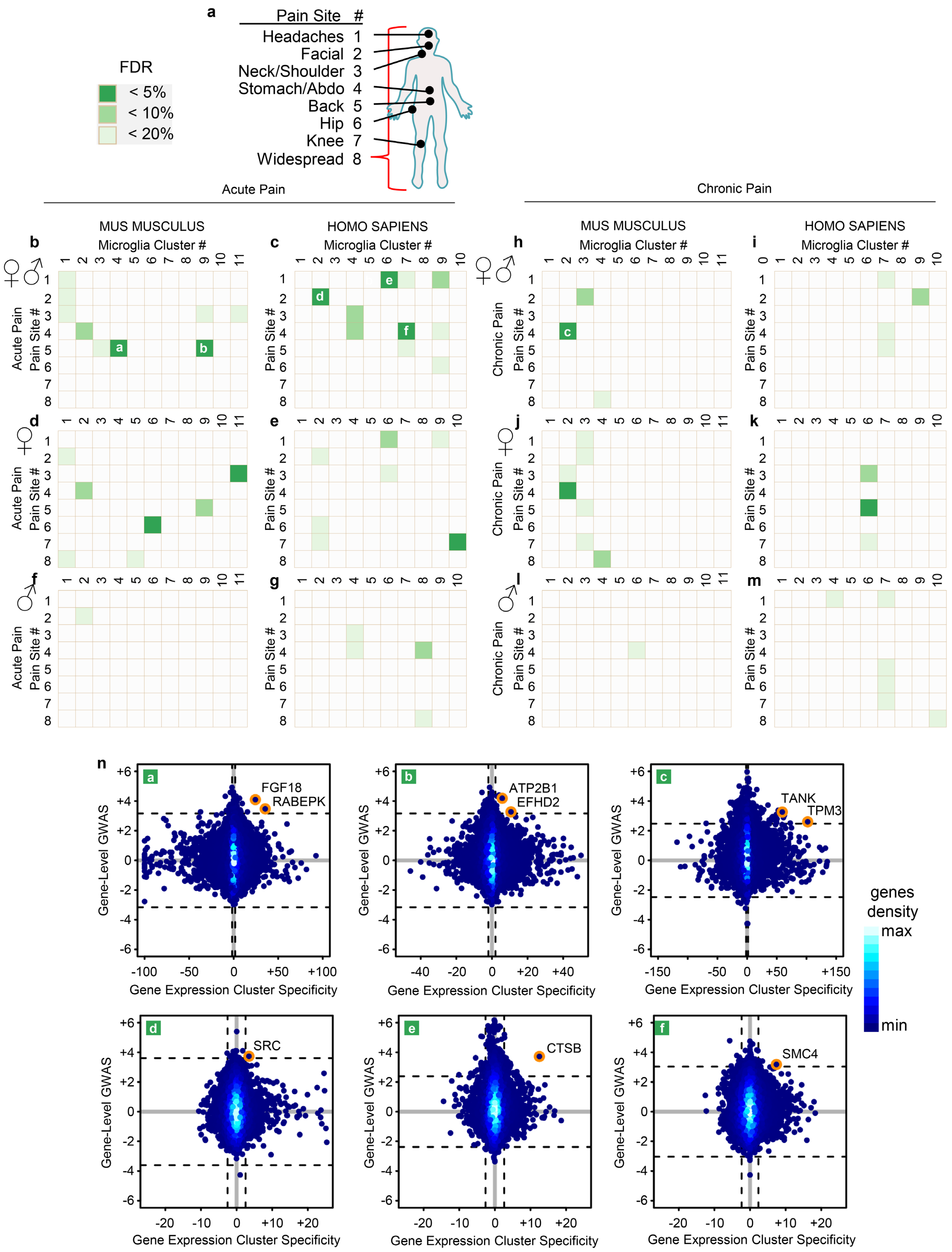
a
Pain Site #
Headaches 1
Facial 2
Neck/Shoulder 3
Stomach/Abdo 4
Back 5
Hip 6
Knee 7
Widespread 8
FDR
< 5%
< 10%
< 20%
Acute Pain
Chronic Pain
MUS MUSCULUS
HOMO SAPIENS
Microglia Cluster #
Acute Pain Pain Site #
Chronic Pain Pain Site #
Pain Site #
n
Gene-Level GWAS
Gene Expression Cluster Specificity
FGF18
RABEPK
ATP2B1
EFHD2
TANK
TPM3
SRC
CTSB
SMC4
genes density
max
min

Supplementary Fig. 12. Contribution of microglia cell type specific transcriptomes to human pain genetics. (a) A diagram showing bodily positions of pain sites considered for GWAS. (**b**-**m**) Contribution of each microglia cell type (cluster #) to each pain site in both sexes (males and females combined), females, and males for acute and chronic pain states. Types of pains are; acute $<$ 1 month, and chronic $>$ 3 months. The contribution is estimated via enrichment of heritability of SNPs in genes principally expressed in specific mouse and human microglia clusters. Heatmaps show stratified, FDR-corrected P-values for enrichment. (**n**) Gene-level contributions. Plotted are test statistics for gene-level GWAS versus gene expression cluster specificity. Top genes are highlighted. Letter in the upper left corner corresponds to heatmap entries of previous panels. Dashed lines indicate FDR 20%. See Supplementary Data 9 for details of the analyses.

Supplementary References

1. Masuda T, *et al.* Spatial and temporal heterogeneity of mouse and human microglia at single-cell resolution. *Nature* **566**, 388-392 (2019).

2. Sankowski R, *et al.* Mapping microglia states in the human brain through the integration of high-dimensional techniques. *Nat Neurosci* **22**, 2098-2110 (2019).

3. Olah M, *et al.* Single cell RNA sequencing of human microglia uncovers a subset associated with Alzheimer's disease. *Nat Commun* **11**, 6129 (2020).

4. Hammond TR, *et al.* Single-Cell RNA Sequencing of Microglia throughout the Mouse Lifespan and in the Injured Brain Reveals Complex Cell-State Changes. *Immunity* **50**, 253-271 e256 (2019).
