# Supplementary material for: Single-cell RNA sequencing reveals time- and sex-specific responses of mouse spinal cord microglia to peripheral nerve injury and links ApoE to chronic pain
Source: Nat Commun. 2022 Feb 11;13:843. doi: 10.1038/s41467-022-28473-8 (PMC8837774; doi:10.1038/s41467-022-28473-8)
Supplement: Supplementary file 3 — Description of Additional Supplementary Files [file 41467_2022_28473_MOESM3_ESM.docx]

**Description of Additional Supplementary Files**

**Title: Supplementary Data 1.**

Description: Unique gene markers for each mouse microglia cluster from Fig. 1e. Average log fold change, p-value, and adjusted p-value are shown (two-sided Wilcoxon rank-sum test with Bonferroni correction).

**Title: Supplementary Data 2.**

Description: Gene markers for each mouse microglia cluster from Fig. 1e. Average log fold change, p-value, and adjusted p-value are shown (two-sided Wilcoxon rank-sum test with Bonferroni correction).

**Title: Supplementary Data 3.**

Description: Differentially expressed genes (DEGs) in SNI versus corresponding sham groups, and sham versus naïve groups, in each microglia cluster at three time points in male and female mice; as well as DEGs when comparing naïve female vs naïve male mice in each microglia cluster. Average log fold change, p-value, and adjusted p-value are shown. Differentially expressed genes between groups were calculated using the Wilcoxon rank-sum test (two-sided) with Bonferroni correction.

**Title: Supplementary Data 4.**

Description: Gene ontology for biological process and molecular function, as well as KEGG enrichment analysis, was conducted using EnrichR^55,56^ (Fisher's exact test, p-values were corrected for multiple testing using the Benjamini-Hochberg method) with gene identities of the top 40 genes either upregulated or downregulated in the DEG comparison SNI vs. sham group in each mouse microglia cluster after removing Rpl and Rps genes. If there were less than 40 genes in any condition, less were considered for the analysis as shown in the analysis description.

**Title: Supplementary Data 5.**

Description: Statistical significance of gene list overlap, using two-sided Fisher's exact test, between DEGs from mouse microglia clusters 1 to 9 at three time points, and microglia transcriptional signature gene lists from Disease-Associated [Microglia](https://www.sciencedirect.com/topics/medicine-and-dentistry/microglia) (DAM), Injury-Responsive Microglia (IRM), and Axon Tract-Associated Microglia (ATM) (Table S2 in ^17^), related to Fig. 3j.

**Title: Supplementary Data** **6.**

Description: Association of APOE-ε2 and APOE-ε4 variants with the report of pain, both acute and chronic, and with and without neuropathic component at several body sites (headaches, facial, neck/shoulders, stomach/abdominal, back, hip, knee, and widespread) in humans in the UK Biobank cohort. The association odd ratios and their P-values were obtained using the haplo.stats R package. Related to Fig. 5.

**Title: Supplementary Data 7.**

Description: Gene markers for each human microglia cluster from Fig. 6a. Average log fold change, p-value, and adjusted p-value are shown (two-sided Wilcoxon rank-sum test with Bonferroni correction).

**Title: Supplementary Data 8.**

Description: Statistical significance of gene list overlap, using two-sided Fisher's exact test, between gene markers from human microglia clusters 1 to 8, and microglia transcriptional signature gene lists from Disease-Associated [Microglia](https://www.sciencedirect.com/topics/medicine-and-dentistry/microglia) (DAM), Injury-Responsive Microglia (IRM), and Axon Tract-Associated Microglia (ATM) (Table S2 in ^17^,), related to Fig. 6d.

**Title: Supplementary Data 9**.

Description: The expression values of the top 1000 highly expressed genes in our human spinal cord microglia dataset and in non-spinal cord (brain) microglia in three publicly available studies: (Masuda et al.,^19^), (Sankowski et al., ^40^), and (Olah et al., ^41^).

**Title: Supplementary Data 10.**

Description: Genome-wide contribution of microglia cell type-specific transcriptomes to human pain genetics. The contribution was estimated using partitioned heritability, which assesses enhanced heritability in GWAS at genetic loci of genes expressed in cell type-specific fashion compared with other, non cell type-specific loci. GWAS for human pain in the UK Biobank were: headaches, facial, neck/shoulder, stomach/abdominal, back, hip, knee, and widespread pain. Pain was defined as acute when present for no more than a month, and chronic when present for three months or more. Partitioned heritability estimates and their P-values were obtained using the LDSC (ldsc.py) computer program. Related to Supplementary Fig. 12b-m.

**Title: Supplementary Data 11.**

Description: Gene-based contribution of microglia cell type-specific transcriptomes to human pain genetics. The contribution was measured using simultaneous cluster-specific expression of genes (X axis) with mean GWAS SNP effect of SNPs in genes (Y axis). Each dot is a gene where tests statistics were tracked. Microglia P-values obtained from logistic regression between cluster ID membership and gene expression level testing cluster specificity of gene expression, while human GWAS P-values obtained from MAGMA computer program, and overall P-value obtained from the product of the microglia and GWAS test statistics. Related to Supplementary Fig. 12n.
