# Supplementary material for: Single-cell RNA sequencing reveals time- and sex-specific responses of mouse spinal cord microglia to peripheral nerve injury and links ApoE to chronic pain
Source: Nat Commun. 2022 Feb 11;13:843. doi: 10.1038/s41467-022-28473-8 (PMC8837774; doi:10.1038/s41467-022-28473-8)
Supplement: Supplementary file 15 — Reporting Summary [file 41467_2022_28473_MOESM15_ESM.pdf]

nature research

Corresponding author(s): Arkady Khoutorsky

Last updated by author(s): January 10, 2022

Reporting Summary

Nature Research wishes to improve the reproducibility of the work that we publish. This form provides structure for consistency and transparency in reporting. For further information on Nature Research policies, see our Editorial Policies and the Editorial Policy Checklist.

Statistics

For all statistical analyses, confirm that the following items are present in the figure legend, table legend, main text, or Methods section.

n/a	Confirmed	
☐	☒	The exact sample size (n) for each experimental group/condition, given as a discrete number and unit of measurement
☐	☒	A statement on whether measurements were taken from distinct samples or whether the same sample was measured repeatedly
☐	☒	The statistical test(s) used AND whether they are one- or two-sided<br>*Only common tests should be described solely by name; describe more complex techniques in the Methods section.*
☐	☒	A description of all covariates tested
☐	☒	A description of any assumptions or corrections, such as tests of normality and adjustment for multiple comparisons
☐	☒	A full description of the statistical parameters including central tendency (e.g. means) or other basic estimates (e.g. regression coefficient) AND variation (e.g. standard deviation) or associated estimates of uncertainty (e.g. confidence intervals)
☐	☒	For null hypothesis testing, the test statistic (e.g. F, t, r) with confidence intervals, effect sizes, degrees of freedom and P value noted<br>*Give P values as exact values whenever suitable.*
☒	☐	For Bayesian analysis, information on the choice of priors and Markov chain Monte Carlo settings
☐	☒	For hierarchical and complex designs, identification of the appropriate level for tests and full reporting of outcomes
☐	☒	Estimates of effect sizes (e.g. Cohen's d, Pearson's r), indicating how they were calculated

Our web collection on statistics for biologists contains articles on many of the points above.

Software and code

Policy information about availability of computer code

Data collection	See below (Data Analysis) for full description of collection software as used for Data analysis.<br>Software for Collection included:<br>Flow cytometry (FACS) for sorting microglia - BD FACS Diva Software 8.0.2<br>Chromium Single Cell Chip (10x Genomics) - Single Cell 3’ Library & Gel Bead Kit v3 (10X Genomics)<br>Illumina Novaseq 6000 /4000 sequencer
Data analysis	Raw sequencing data for each sample was converted to matrices of expression counts using the Cell Ranger software provided by 10X Genomics (version 3.0.2). Briefly, raw BCL files from the Illumina HiSeq were demultiplexed into paired-end, gzip-compressed FASTQ files using Cell Ranger’s mkfastq. Using Cell Ranger’s count, reads were aligned to the GRCm38 (mm10) mouse reference genome, and transcript counts quantified for each annotated gene within every cell. The resulting UMI count matrices (genes × cells) were then provided as input to Seurat suite (version 3.1.0).<br>GraphPad Prism 9.3.0 was used for data analysis and presentation, EnrichR and gProfiler for GO, ImageJ (1.53f51) and Photoshop (22.1.0) for image processing.

For manuscripts utilizing custom algorithms or software that are central to the research but not yet described in published literature, software must be made available to editors and reviewers. We strongly encourage code deposition in a community repository (e.g. GitHub). See the Nature Research guidelines for submitting code & software for further information.

Data

Policy information about availability of data

All manuscripts must include a data availability statement. This statement should provide the following information, where applicable:

- Accession codes, unique identifiers, or web links for publicly available datasets
- A list of figures that have associated raw data
- A description of any restrictions on data availability

Single-cell RNA-sequencing data generated in this study have been deposited in the Gene Expression Omnibus under the accession GSE162807 (https://www.ncbi.nlm.nih.gov/geo/query/acc.cgi?acc=GSE162807). Publicly available datasets generated in previous scRNA-seq studies (Masuda et al., accession code GSE:124335, Sankowski et al., accession code GSE:135437, and Olah et al., raw count matrix provided by the authors) were used in this work.
Cell Ranger (v.3.0.1) (10X Genomics) was used for sample demultiplexing, barcode processing, unique molecular identifiers filtering, gene counting, and sample mapping to the reference transcriptome (mouse mm10 v.1.2.0).
UMI count data for neurons in the mouse dorsal horn were obtained from the GEO data repository (accession number GSE103840).
Correspondence between human gene symbols (HGNC) and mouse ones (MGI) was established using the BioMart R package

Field-specific reporting

Please select the one below that is the best fit for your research. If you are not sure, read the appropriate sections before making your selection.

- [x] Life sciences
- [ ] Behavioural & social sciences
- [ ] Ecological, evolutionary & environmental sciences

For a reference copy of the document with all sections, see nature.com/documents/nr-reporting-summary-flat.pdf

Life sciences study design

All studies must disclose on these points even when the disclosure is negative.

Sample size	The sample sizes were selected based on published studies in the field (e.g. Peirs et al, Neuron, 2020; Martin et al, JCI, 2017; Yuanyuan Liu et al, Nature, 2018; Gang Chen et al, Nat. Neuroscience, 2017). For histology, sample sizes (3-4 mice) were used. 8-12 mice were used for behavioral studies.
Data exclusions	We did not exclude any data from the dataset (no animals or data points were excluded from the analyses).
Replication	All conclusions described in the paper were confirmed by analysis of indicated biological replicates, and all attempts of replication were successful. The main results described in Figs 2f-i and 4f-g were replicated in two independen experiments, all other experimetns were performed once.
Randomization	For all experiments, including molecular and behavioral studies, mice were randomly assigned to control and experimental groups.
Blinding	Blinding was done on all imaging and behavioural studies. File names of images were recoded for blinding purposes and unblinding was done after quantification was complete. FACS, cell capture and subsequent processing for sequencing were performed blindly as well.

Reporting for specific materials, systems and methods

We require information from authors about some types of materials, experimental systems and methods used in many studies. Here, indicate whether each material, system or method listed is relevant to your study. If you are not sure if a list item applies to your research, read the appropriate section before selecting a response.

Materials & experimental systems

n/a	Involved in the study	
[ ]	[x]	Antibodies
[x]	[ ]	Eukaryotic cell lines
[x]	[ ]	Palaeontology and archaeology
[ ]	[x]	Animals and other organisms
[ ]	[x]	Human research participants
[x]	[ ]	Clinical data
[x]	[ ]	Dual use research of concern

Methods

n/a	Involved in the study	
[x]	[ ]	ChIP-seq
[ ]	[x]	Flow cytometry
[x]	[ ]	MRI-based neuroimaging

Antibodies

Antibodies used	(1:500 Ki67 [Abcam, ab15580], 1:500 Iba1 [Synaptic systems, 234 004], 1:500 Iba1 [Wako, 019-19741], 1:500 ApoE [Cell Signaling Technology, 13366]), 1:500 NeuN [Abcam, ab104224] and 1:500 GFAP [Abcam, ab4674]). Secondary antibodies (1:500 Goat anti-guinea pig Alexa Fluor 647 [Thermo Fisher Scientific, A-21450], 1:500 Donkey anti-rabbit Alexa Fluor 568 [Thermo Fisher Scientific.

	A10042], 1:500 Goat anti-guinea pig Alexa Fluor 488 [Thermo Fisher Scientific, A-11073], 1:500 Goat anti-mouse Alexa Fluor 405 [Thermo Fisher Scientific, A-31553], 1:500 Goat anti-chicken Alexa Fluor 647 [Thermo Fisher Scientific, A-32933])<br><br>FACS antibodies:<br>CD11b PE (Biolegend, 101208, 1:200)<br>CD45 APC/Cy7 (Biolegend, 103116, 1:200)<br>CX3CR1 APC (Biolegend, 149008, 1:200)<br>DAPI (1:10000)
Validation	The following antibodies were validated by the supplier and were chosen based on their use in the literature.<br><br>FACS antibodies:<br>CD11b PE (Biolegend, 101208)<br>CD45 APC/Cy7 (Biolegend, 103116)<br>CX3CR1 APC (Biolegend, 149008)<br><br>Immunohistochemistry antibodies:<br>Ki67 (Abcam, ab15580)<br>Iba1 (Synaptic systems, 234 004)<br>Iba1 (Wako, 019-19741)<br>ApoE (Cell Signaling Technology, 13366)<br>NeuN (Abcam, ab104224)<br>GFAP (Abcam, ab4674)<br><br>Literature;<br>Lacoste, B., et al. Sensory-related neural activity regulates the structure of vascular networks in the cerebral cortex. Neuron 83, 1117-1130 (2014).<br>Linker, K.E., et al. Microglial activation increases cocaine self-administration following adolescent nicotine exposure. Nature Communications 11, 306 (2020).<br>Keren-Shaul, H., et al. A Unique Microglia Type Associated with Restricting Development of Alzheimer's Disease. Cell 169, 1276-1290 e1217 (2017).<br>Hammond, T.R., et al. Single-Cell RNA Sequencing of Microglia throughout the Mouse Lifespan and in the Injured Brain Reveals Complex Cell-State Changes. Immunity 50, 253-271 e256 (2019).<br>Tsai, P.-Y., et al. IL-22 Upregulates Epithelial Claudin-2 to Drive Diarrhea and Enteric Pathogen Clearance. Cell Host & Microbe 21, 671-681.e674 (2017).<br>Jaudon, F., et al. Kidins220/ARMS controls astrocyte calcium signaling and neuron–astrocyte communication. Cell Death & Differentiation 27, 1505-1519 (2020).<br>Buttini, M., et al. Modulation of Alzheimer-Like Synaptic and Cholinergic Deficits in Transgenic Mice by Human Apolipoprotein E Depends on Isoform , Aging, and Overexpression of Amyloid β Peptides But Not on Plaque Formation. J Neurosci 22, 10539-10548 (2002).

Animals and other organisms

Policy information about studies involving animals; ARRIVE guidelines recommended for reporting animal research

Laboratory animals	Female and male 2-8 month-old mice on C57BL/6 (B6) genetic background were used. Reporter Ai14 tdTomato mice (The Jackson laboratory, stock #007914) were crossed with TMEM119CreERT2 mice (the Jackson laboratory stock #031820) to generate TdTomato;TMEM119CreERT2 animals and used at 2-8 months of age. Mice were housed at McGill University animal facility in standard shoebox cages (5 per cage) and maintained at a temperature-controlled environment on a 12:12 h light/dark cycle (lights on at 07:00 h). Food (Envigo Teklad 8604, Lachine, QC, Canada) and water were provided ad libitum.
Wild animals	Wild animals were not used in this study.
Field-collected samples	Field-collected samples were not used in this study.
Ethics oversight	All mouse experiments were approved by the Animal Care Committee at McGill University (protocol number 2016-7869, Arkady Khoutorsky) and complied with Canadian Council on Animal Care guidelines.

Note that full information on the approval of the study protocol must also be provided in the manuscript.

Human research participants

Policy information about studies involving human research participants

Population characteristics	Analyses were restricted to the “White British” ancestry, after genotyping quality control performed by the UKB detailed in the resource: https://biobank.ctsu.ox.ac.uk/crystal/crystal/docs/genotyping_qc.pdf. Age, age squared, sex, recruitment sites, genotyping array, and first 40 principal genetic components were used as co-variables.

Recruitment	Analysis was performed on pre-existing data from the UK Biobank.
Ethics oversight	The meta-analysis work to combine all studies was pre-approved by RTI International Institutional Review Board. Each individual study was approved by IRBs at their respective sites. The current study was conducted under UK Biobank application no. 20802, application courteously initiated by Dr. Samar Khoury.<br>Human spinal cord tissue was harvested from organ donors through a collaboration with Transplant Quebec. All procedures are approved by and performed in accordance with the ethical review board at McGill University (IRB#s A04-M53-08B).

Note that full information on the approval of the study protocol must also be provided in the manuscript.

Flow Cytometry

Plots

Confirm that:

- [x] The axis labels state the marker and fluorochrome used (e.g. CD4-FITC).
- [x] The axis scales are clearly visible. Include numbers along axes only for bottom left plot of group (a 'group' is an analysis of identical markers).
- [x] All plots are contour plots with outliers or pseudocolor plots.
- [x] A numerical value for number of cells or percentage (with statistics) is provided.

Methodology

Sample preparation	For flow cytometry sorting samples were processed the same as described in the methods section. They were stained for CD45/CD11b/CX3CR1 and subsequently rinsed with FACS buffer. Prior to running samples, compensations were calculated with unstained controls (CD45 negative) and compensation beads (BD).
Instrument	Samples were sorted using either a FACSAria III cell sorter equipped with 405 nm, 488 nm and 640 nm lasers and the appropriate filters or a FACSAria Fusion equipped with a 405 nm, 488 nm, 561 nm and 633 nm lasers and the appropriate filters (both from BD Biosciences)
Software	BD FACSDiva 8.0.2
Cell population abundance	Microglia represented 13.3% of all events. Microglia were sorted based off of the gating strategy detailed below. Positive fractions (CD45low/CD11b+/CX3CR1+) were taken for single cell sequencing, and bio-informatic analysis was used to assess expression levels of canonical markers of microglia for confirmation of identity.
Gating strategy	Sorting microglia for Single-Cell RNA Sequencing: Cells were gated based on size using Forward and Side scatter, followed by identification of singlets using FSC-H and FSC-A. After gating on Live cells, we gated on CD45 low/CD11b+/CX3CR1+ were sorted for sequencing.<br>Positive fraction was sorted for single cell sequencing.

- [x] Tick this box to confirm that a figure exemplifying the gating strategy is provided in the Supplementary Information.
